# Response to the Letter to the Editor: The Potential Consideration of Implicit/Unconscious Gender Bias in Clinical Management Among Patients With Familial Hypercholesterolemia

**DOI:** 10.1002/hsr2.70996

**Published:** 2025-07-07

**Authors:** Ngoc‐Thanh Kim, Doan‐Loi Do, Mai‐Ngoc Thi Nguyen, Thanh‐Tung Le, Hong‐An Le, Thanh‐Huong Truong

**Affiliations:** ^1^ Department of Cardiology Hanoi Medical University Hanoi Vietnam; ^2^ Vietnam National Heart Institute Bach Mai Hospital Hanoi Vietnam; ^3^ Faculty of Medicine Phenikaa University Hanoi Vietnam

To the Editor,

Our recent article examined the significant sex‐based disparities observed in index‐case with familial hypercholesterolemia (FH) in Vietnam [1]. We appreciated the insightful letter addressing the persistent implicit/unconscious gender bias (IGB) that contributes to healthcare disparities.

Sex‐based disparities in clinical profiles and treatment have also been documented in the global registry of the European Atherosclerosis Society—Familial Hypercholesterolemia Studies Collaboration [2]. FH women frequently encounter delays in diagnosis and receive inadequate lipid‐lowering therapy compared to men, despite having comparable cholesterol levels. Notably, men often present with more classic FH phenotypes, with the incidence of premature coronary artery disease (CAD) approximately twofold higher than that in women. This disparity may facilitate earlier diagnosis of FH in men, as clinicians may be more inclined to consider FH in the context of male patients presenting with cardiovascular symptoms.

The observed differences in the incidence of premature CAD between genders in our study may also be related to lifestyle factors such as smoking, with up 76.2% of male FH patients identified as smokers, while none of the female patients reported smoking [1]. According to the 2019 ESC/EAS Guidelines for the Management of Dyslipidemias, patients with CAD should aim for LDL‐C levels of < 1.4 mmol/L, which typically necessitating the use of high‐intensity statins [3]. This guideline elucidates the sex‐based disparities in treatment, where 36.5% of FH male patients receive high‐intensity statin therapy, comparing to only 10.6% of women, reflecting the higher prevalence of CAD in male FH patients within our study cohort. It is essential to recognize FH as a specific and severe form of dyslipidemia, rather than a generalized condition, ensuring that these patients receive appropriate lipid‐lowering therapy. Before experiencing cardiovascular event, many FH patients may merely be classified as having hypercholesterolemia, rendering them less likely to meet the guideline‐recommended LDL‐C targets for high‐risk individuals.

Thus, IGB may significantly impact the clinical care for FH patients. Identifying these imbalances will enable us to dismantle barriers to equitable care through targeted educational initiatives and enhanced training for clinicians. Furthermore, fostering real‐time information exchange within structured peer networks can improve clinical accuracy, and reduce IGB in diagnosis and treatment [4]. We strongly advocate for further analyses of gender difference, including the implications of IGB, to refine life course management strategies for FH patients [5].

## Author Contributions

**Ngoc‐Thanh Kim:** conceptualization, investigation, writing – original draft, methodology, software, formal analysis, project administration, visualization, validation. **Doan‐Loi Do:** writing – original draft, funding acquisition, investigation, conceptualization, methodology. **Mai‐Ngoc Thi Nguyen:** conceptualization, investigation, writing –original draft, methodology. **Thanh‐Tung Le:** writing – original draft, funding acquisition, investigation, conceptualization, visualization, validation, methodology, project administration, resources, data curation, supervision. **Hong‐An Le:** conceptualization, investigation, writing – original draft, methodology, validation. **Thanh‐Huong Truong:** conceptualization, investigation, funding acquisition, writing – original draft, methodology, validation, visualization, software, formal analysis, project administration, resources, data curation, supervision.

## Conflicts of Interest

The authors declare no conflicts of interest.

## Transparency Statement

T.‐H.T. and N.‐T.K. affirm that this manuscript is an honest, accurate, and transparent account of the study being reported; that no important aspects of the study have been omitted; and that any discrepancies from the study as planned (and, if relevant, registered) have been explained.

## Data Availability

Data sharing is not applicable to this article as no new data were created or analyzed in this study.

## References

[1] N. T. Kim , D. L. Do , M. N. T. Nguyen , T. T. Le , H. A. Le , and T. H. Truong , “Sex‐Based Disparities in Index Cases of Familial Hypercholesterolemia in Vietnam: A Cross‐Sectional Study,” Health Science Reports 8, no. 3 (2025): e70579. 40124921 10.1002/hsr2.70579 PMC11925809

[2] EAS‐FHSC ., “Global Perspective of Familial Hypercholesterolaemia: A Cross‐Sectional Study From the EAS Familial Hypercholesterolaemia Studies Collaboration (FHSC),” The Lancet 398, no. 10312 (2021): 1713–1725. 10.1016/S0140-6736(21)01122-3 34506743

[3] F. Mach , C. Baigent , A. L. Catapano , et al., “2019 ESC/EAS Guidelines for the Management of Dyslipidaemias: Lipid Modification to Reduce Cardiovascular Risk,” European Heart Journal 41, no. 1 (2020): 111–188. 31504418 10.1093/eurheartj/ehz455

[4] D. Centola , D. Guilbeault , U. Sarkar , E. Khoong , and J. Zhang , “The Reduction of Race and Gender Bias in Clinical Treatment Recommendations Using Clinician Peer Networks in an Experimental Setting,” Nature Communications 12, no. 1 (2021): 6585. 10.1038/s41467-021-26905-5 PMC8593068 34782636

[5] S. S. Gidding , D. J. Blom , B. McCrindle , et al., “Life Course Approach for Managing Familial Hypercholesterolemia,” Journal of the American Heart Association 14, no. 7 (2025): e038458. 40118807 10.1161/JAHA.124.038458 PMC12132838

